# Effect of subthalamic and nigral deep brain stimulation on speech and voice in Parkinson’s patients

**DOI:** 10.1007/s00702-024-02860-5

**Published:** 2024-11-28

**Authors:** Frank Müller, Julie Cläre Nienstedt, Carsten Buhmann, Ute Hidding, Alessandro Gulberti, Monika Pötter-Nerger, Christina Pflug

**Affiliations:** 1 Department of Voice, Speech and Hearing Disorders, Center for Clinical Neurosciences, University Medical Center Hamburg-Eppendorf, 20246 Hamburg, Germany; 2 Department of Neurology, University Medical Center Hamburg-Eppendorf, 20246 Hamburg, Germany

**Keywords:** Speech, Voice, DBS, STN, SNr, Parkinson’s disease

## Abstract

Deep brain stimulation can influence the speech and voice quality in Parkinson´s disease (PD). This controlled, randomized, double-blind, cross-over clinical trial was conducted in 15 PD patients with bilateral subthalamic deep brain stimulation (DBS) to compare the effects of STN-DBS with combined subthalamic and nigral stimulation (STN + SNr-DBS) and DBS OFF on speech and voice parameters in PD patients. Speech and voice were analyzed subjectively using questionnaires (voice/pronunciation quality VAS, VHI, SHI) and objectively using audio analysis (maximum phonation time, AVQI, mean F0, intonation, syllable rate, reading time). Both stimulation conditions, STN + SNr-DBS and STN-DBS, revealed heterogeneous effects on speech and voice production with a slight beneficial effect on the voice quality of individual patients compared to DBS OFF, but not in the whole group. Small, but not significant effects were seen only in subjective voice quality on the VAS and intonation (both stimulation conditions compared to DBS OFF). No significant changes of the objective speech parameters during the audio analysis could be observed (both stimulation conditions compared to DBS OFF). There were no significant differences between STN + SNr-DBS and STN-DBS in any speech and voice domain. The beneficial effects on speech and voice production are minor in most patients compared to the motor improvements by DBS. Both STN-DBS and STN + SNr-DBS were safe, with comparable effects between both DBS modes, and represent no contraindications from the perspective of the voice specialist.

**Supplementary Information:**

The online version contains supplementary material available at 10.1007/s00702-024-02860-5.

## Introduction

Up to 90% of people with Parkinson’s disease (PD) develop speech and voice disorders during the disease course (Dashtipour et al. 2018), which negatively impact their functional communication and quality of life (Miller et al. 2006). The characteristic speech pattern of PD is hypokinetic dysarthria, classically characterized by reduced loudness, reduced prosodic contour or emphasis, short pauses and/or rapid speech tempo, imprecise articulation, and breathy or hoarse voice (Ho et al. 1999; Schulz and Grant 2000). Several parts of speech production may be affected, including respiration, laryngeal function, resonance, articulation, and prosody (Pinto et al. 2004).

In addition to predominantly dopaminergic drug treatment, stereotactic implantation of electrodes for high-frequency deep brain stimulation (DBS) in the subthalamic nucleus (STN) has been established in recent years as a therapy for PD, showing good results in the improvement of drug-resistant tremor, motor fluctuations, dyskinesias and quality of life (Deuschl et al. 2006, 2013; Schuepbach et al. 2013). Although motor performance is optimized during DBS surgery, in many PD patients, speech impairment must be accepted as a compromise and is perceived by a third of patients as their greatest limitation (Dashtipour et al. 2018).

Recently, simultaneous stimulation of the subthalamic nucleus and substantia nigra (STN + SNr stimulation) has been introduced in PD patients with the primary goal to improve another axial symptom, freezing of gait (Weiss et al. 2013). So far, the effect of STN + SNr-DBS on speech and voice is unknown, although this stimulation condition might be of particular interest considering neuroanatomical networks involved in speech generation. In a proposed model summarizing data derived from lesion, stimulation, single-unit recording and brain imaging studies in animal models and humans it was hypothesized that voluntary control of vocalizations is mediated by the motor cortex via “direct” pyramidal projections as well as “indirect” cortical downstream pathways via basal ganglia including projections to the putamen, substantia nigra, parvocellular reticular formation to phonatory motoneurons (Jürgens 2002). Non-verbal vocalizations are also mediated by projections from the anterior cingulate gyrus and adjacent mesiofrontal areas to cranial nerve motor nuclei in the lower brainstem via midbrain structures as the periaqueductal grey and adjacent tegmentum (Ackermann 2008), which are modulated by SNr projections. The tegmentum and reticular formation represent an important convergence zone of orofacial premotor neurons, proposed to be involved in coordination of phonatory muscles (Jürgens 2002). Thus, one could assume, that speech production might be particularly ameliorated by nigral costimulation due to strong interconnections of nigral neurons and reticular phonatory premotor neurons. On the other hand, the combined stimulation of STN + SNr is associated with an increased VTA (volume of tissue activated) with a risk of unintentional costimulation of the lateral capsula interna resulting in deterioration of speech. It is therefore of interest, in which direction speech is impacted by the combined STN + SNr-DBS.

Heterogeneous results on the effects on speech and voice quality have been reported in the literature for both drug and DBS in different target areas as the globus pallidus, STN-DBS or other nuclei as thalamic or zona incerta regions in PD (Baudouin et al. 2023). For example, in a meta-analysis, 9.3% of PD patients treated with STN-DBS were found to have stimulation-related dysarthria (Kleiner-Fisman et al. 2006), whereas another cohort study showed improvements in voice quality and prosody (Skodda et al. 2014).

The aim of the current study was to assess the impact of combined STN + SNr stimulation on speech and voice in PD and to compare the effect with conventional STN stimulation.

## Methods

### Study design

This controlled, randomized, double-blind, cross-over clinical trial was conducted at the University Medical Center Hamburg-Eppendorf and compares the effect of STN-DBS and combined STN + SNr-DBS as described previously (Hidding et al. 2017; Pflug et al. 2020). The study was approved by the local ethics committee and was conducted in agreement with the Code of Ethics of the World Medical Association (Declaration of Helsinki, 1967). Written informed consent was obtained from all participants. Patients were examined by a non-blinded therapeutic movement disorder specialist, who programmed the stimulation conditions, and by a blinded phoniatric examiner at three visits each three weeks apart. All visits were performed in medication-on condition.

### Stereotactic procedures

Detailed description of DBS surgery for optimal target site localization for electrode implantation by MR-based stereotactic planning, microrecording and stimulation has been described previously (Steigerwald et al. 2008). Electrode position at the upper border of the SNr was defined as location of the electrode tip at least 4.5–6 mm inferior to AC-PC line. The position of the implanted electrodes (model 3389; Medtronic, Minneapolis, Minnesota, USA, in 8 cases, and 8-poled electrode model, Boston Scientific, Valencia, CA, USA, in 2 cases) was determined by coregistration of the preoperative T1-MRI-scans and post-operative CT-scans performed with Brainlab (iPlan software; Brainlab, Feldkirchen, Germany).

### DBS settings

After testing thresholds for side effects in the SNr, defined stimulation settings for the STN and STN + SNr stimulation were fixed for the course of the experiment. The STN settings did not differ from those before the study in everyday life condition. Afterwards, stimulation of the STN or combined STN + SNr was set in a randomized manner for the following 3 weeks. Speech and voice examinations were performed in the following order:

Firstly, patients were investigated at baseline visit with DBS switched off (STIM-OFF) mode.

(I) In phase I, patients were examined after random assignment to one of two DBS modes: conventional STN-DBS or combined STN + SNr-DBS.
(II) After 3 weeks, reprogramming was performed in a cross-over procedure for the following 3 weeks. In this phase II, the third evaluation of the patients occurred as described before (Pflug et al. 2020).

Patients were blinded for their stimulation condition (conventional STN stimulation or combined STN + SNr stimulation). At the end, patients were unblinded and the preferred stimulation condition was programmed as permanent therapeutic stimulation. Medication and stimulation parameters were held constant during the phase I and II of the study. Only in one case, the stimulation amplitude in the SNr had to be reduced after 2 days due to dyskinesias.

### Subjects

15 patients suffering from PD participated in the study. Four patients withdrew from the study due to side effects of combined STN + SNr-DBS. Side effects of nigral costimulation were worsening of motor functions as well as a lack of beneficial effects of levodopa, akathisia, general uncomfortable feeling, aggressiveness, and increased confusion and hallucinations during chronic stimulation. The results of the 11 patients, who completed the full study protocol, were considered. Demographic and clinical characteristics are described in detail in Table S1 and in a previous publication (Pflug et al. 2020). The gender distribution of male:female was 10:1 and the mean age was 63.5 years (range 53–74). Mean disease duration was 12.0 ± 5.0 years and mean Hoehn & Yahr stage 2.2 ± 0.36.

All patients had a bilateral STN-DBS with the deepest contacts of the electrodes within the dorsal aspects of the SNr along image-based electrode reconstruction (> 4.5 mm below AC-PC). The study inclusion and exclusion criteria, as well as the selection criteria for DBS surgery, have been reported in detail previously (Hidding et al. 2017). The location of the electrode contacts was controlled by stereotactic coordinates based on MR imaging and intraoperative microrecording. The mean stereotactic coordinates of the ventral most DBS contact relative to the mid-commissural point of the 11 PD patients (mean ± standard deviation in mm) were x = 10.4 ± 0.9, y = 2.8 ± 1.3, z = 6.3 ± 1.0 for the left hemisphere and x = 10.1 ± 1.7, y = 2.7 ± 1.5, z = 5.7 ± 1.4 for the right side (x = lateral to midline, y = posterior to MCP, z = inferior to AC-PC level, see Fig. S1 in the Supplement).

### Assessments and speech and voice analysis

To evaluate the effects of a combined STN + SNr stimulation on speech and voice function and the patients´ self-perception, clinical examination and speech and voice analysis were performed.

Since there are no standards or guidelines for voice diagnostics in PD, this study partially followed the protocol of Rusz et al. (Rusz et al. 2020). Speech and voice were analyzed subjectively using questionnaires and objectively using audio analysis.

### Subjective evaluation of speech and voice

All patients completed the validated questionnaires *Speech Handicap Index (SHI)* (Rinkel et al. 2008) and *Voice Handicap Index (VHI)* (Rosen et al. 2004), each in the German language version. The SHI and VHI are standardized questionnaires validated in many languages to measure the impact of speech and voice problems on a patient's quality of life (low values indicate a lower handicap; items relate to shame, effort, stress, avoidance, anger).

In addition, perceived voice quality (a) and pronunciation quality (b) on that day were assessed on a 10 cm visual analog scale (VAS) by the patients. A score below 30% on the VAS was classified as acceptable voice and pronunciation quality in the respective case.

### Objective evaluation of speech and voice

*Vocal tasks* The speech and voice analysis consisted of three vocal tasks, namely a reading passage, a sustained vowel, and sequential and alternating motion rates.

- Sustained vowel: the patient produces vowel [a] at comfortable pitch and loudness as long as possible, max. two repetitions to improve the result.
- Reading passage: the patient reads the short standard text "The North Wind and the Sun" by Aesop (in German—"Nordwind und Sonne”) in a comfortable pitch and loudness.
- Sequential and alternating motion rates (diadochokinesis): the patient produces syllables [pa] and [pataka] as fast as possible on one breath with max. two repetitions to improve each result.

In all patients, the three diagnostic tasks were performed in all three conditions, STIM-OFF, Phase I, and Phase II with respective STN-DBS or STN + SNr-DBS, respectively.

### Audio recording and processing

The tasks were recorded using the SpeechStudio system (Laryngograph Ltd., London). This was done in a pseudo-random order to avoid bias due to decreasing patient endurance. The signal-to-noise ratio of background noise in the room and voice loudness was checked to be > 30 dB, as recommended by Deliyski et al. (2005). The sample rate was 16 kHz, the amplitude resolution was 16 bits. The system records the microphone signal at 30 cm distance to the mouth of the examined person in uncompressed WAV format. The optimal recording level was set manually during a test recording.

The Praat system, version 6.1.38 (Boersma and Weenink 2021), was used for all audio processing.

The *mean fundamental frequency (F0)*, the main contributor to auditory perception of vocal pitch, was extracted from the standard text recording by the Praat function “To pitch…” (automatic time step, pitch floor/ceiling at 75/300 Hz) and the Praat function “Get mean…” and measured in Hertz (Hz). As a measure of speech intonation, the *F0 variation coefficient* was calculated from the quotient of F0 standard deviation (Praat function “Get standard deviation…”) and mean fundamental frequency.

The *maximum syllable rate* for [pa] and [pataka] was measured using a digital Praat program script (Mayer 2021) and adjusted based on the Praat sound intensity function “Get value in frame…”. The script suggested the most likely syllable boundaries which had to be confirmed by visual inspection of the boundaries in a spectrogram plot. The script determined the rate value for each task by extracting the mean rate of the fastest 5 subsequent syllables.

The *reading time (RT)* was measured manually from the task “reading passage” analogous to the MPT in the plot of the time signal.

*The maximum phonation time (MPT)* for the “sustained vowel” was measured as the duration of vowel [a] produced on one breath. The MPT will be reduced in persons with reduced lung capacity or disturbed voice. A critical threshold was set to 12 s from clinical experience, but without evidence from literature, as the parameter suffers from many side effects (Baken and Orlikoff 2000).

For the acoustic measure of voice quality, the *Acoustic Voice Quality Index (AVQI)* value (Maryn et al. 2010) was determined by the corresponding Praat script with version 03.01 and following the procedure described by Barsties et al. (2018). It uses 3 s of the sustained vowel sound and the first 27 syllables of the reading passage (Barsties von Latoszek et al. 2020), which in this study had a mean length of 6.8 s. The AVQI combines several acoustic parameters to one index, which shows bigger values for disturbed (hoarse) voices. The threshold for distinction between normophonic and dysphonic was set to 1.85, according to Barsties et al. (2020) for German language.

### Correlation with TEED

 All speech and voice parameters were correlated with the total electrical energy delivered by DBS (TEED). This parameter is calculated from the DBS stimulation parameters. It is the product of the (1) squared impulse amplitude, (2) the impulse width, and (3) the impulse frequency as described by Moreau (Moreau et al. 2008).

### Statistics

 Group differences were tested with the t-test for connected samples. The significance level was set to 0.05.

## Results

For all patients the TEED (in µW) was higher with STN + SNr stimulation (211 ± 74) than with STN stimulation (155 ± 57). The correlation of TEED to the stimulation condition is very strong (0.85) and the mean values are significantly different (p = 0.001, CI: − 82.9, − 29.8, t test for connected samples). The exploratory, uncorrected correlation analysis for TEED with speech and voice parameters revealed very weak connections and is reported for intonation only.

The comparison of the stimulation conditions (conventional STN-DBS versus combined STN + SNr-DBS) showed no significant effects related to the mean values of speech and voice parameters. A more detailed look at the individual subjects revealed relevant deviations, but only for isolated parameters.

### Objective speech analysis (mean F0, intonation, syllable rate, reading time)

#### Mean fundamental frequency (F0) (male patients only)

**Fig. 1:**
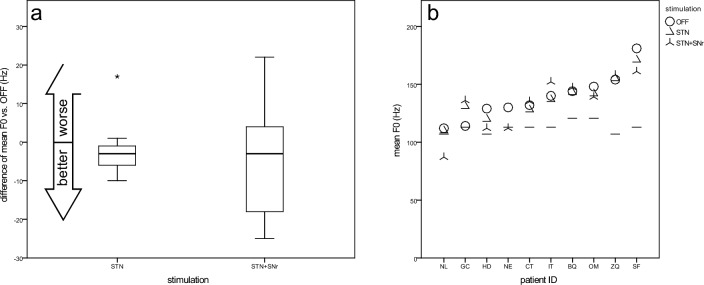
Mean fundamental frequency (F0)—(**a**) boxplots of changes from STIM-OFF for stimulation groups and **b** scatterplot for individual patient outcomes (sorted by values for STIM-OFF). Short lines indicate age and gender matched reference values (conversational voice) (Berg et al. 2017)

Because of differences in mean fundamental frequency in speaking voice between women and men, the only female patient was excluded for analysis of F0. Interestingly, most male patients showed a higher baseline mean frequency (F0) than the matched reference value (solid lines in Fig. 1b) for the reading passage, regardless of the stimulation condition. Both stimulation conditions had no relevant impact on mean fundamental frequency: OFF (138 ± 19), STN (142 ± 22), STN + SNr (139 ± 28) (Fig. 1a).

#### Intonation (variation coefficient of F0, male patients only)

**Fig. 2:**
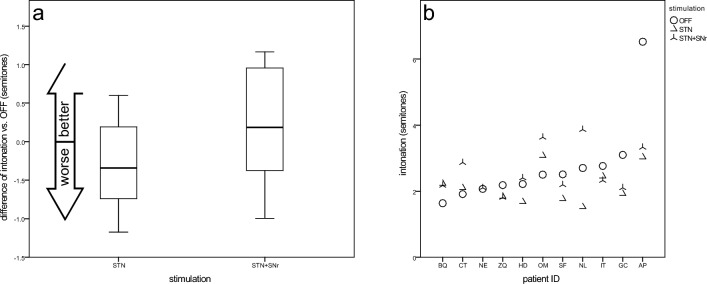
Variation coefficient of F0 as a measure for intonation—**a** boxplots of changes from STIM-OFF for stimulation groups and **b** scatterplot for individual patient outcomes (sorted by values for STIM-OFF)

Compared to OFF mode (2.4 ± 0.45), mostly the intonation was reduced under STN-DBS (2.1 ± 0.47), and slightly enhanced under STN + SNr-DBS (2.6 ± 0.71) (Fig. 2a). Seven patients showed improved intonation at least under one stimulation, four patients showed reduced intonation under both stimulations (Fig. 2b). The mean intonation values of STN-DBS are smaller than those of the STN + SNr-DBS, but do not reach statistical significance (p = 0.07, CI: − 0.06, 1.1, t test for connected samples). Patients with STN + SNr stimulation have a tendency to benefit from a better intonation.

The correlation graph of the intonation with TEED (Suppl. Fig. S2) shows two distinct areas: STN stimulation dominates the low energy stimulation area. The improved intonation under STN + SNr stimulation compared to STN alone, is achieved by a higher stimulation energy.

#### Syllable rates for [pataka], [pa], and reading time

The results are shown in the supplement.

### Objective voice analysis (MPT, AVQI)

#### Maximum phonation time (MPT)

**Fig. 3:**
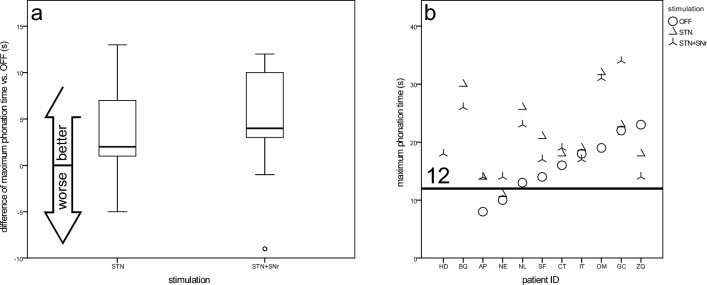
Maximum phonation time—**a** boxplots of changes from STIM-OFF for stimulation groups and **b** scatterplot for individual patient outcomes (sorted by values for STIM-OFF). Values above the line (12 s) are regarded as physiological

All but one patient had an adequate maximum phonation time of at least 12 s under both DBS modes. Eight patients improved MPT under at least one stimulation, only one patient had worse results under both stimulation conditions (Fig. 3b). In general, STN-DBS (20.2 ± 6.3) and STN + SNr-DBS (20.3 ± 7.5) lead to higher MPT results than STIM-OFF (15.9 ± 5.1) (Fig. 3a), but both stimulation conditions were not significantly different to OFF (STN CI: − 8.9, 0.3; STN + SNr CI: − 9.6, 0.7, *t* test for connected samples).

#### Acoustic voice quality index (AVQI)

**Fig. 4:**
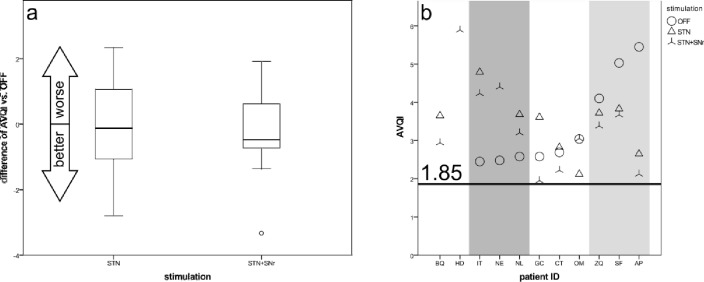
AVQI—**a** boxplots of changes from STIM-OFF for stimulation groups and **b** scatterplot for individual patient outcomes (sorted by values for STIM-OFF). Solid line: threshold for normophonic voices

The AVQI results showed that all patients are outside the normophonic range regardless of DBS mode (Fig. 4b). The average values of the three modes were quite similar (DBS OFF (3.4 ± 1.2), STN-DBS (3.4 ± 0.8), STN + SNr-DBS (3.4 ± 1.2)). At the individual level, STN + SNr-DBS resulted in higher AVQI values compared to STN-DBS in all cases except one. Patients with low AVQI values in the DBS OFF tended to have higher values under stimulation and vice versa. Overall, there is no clear group trend. Some patients improved AVQI values under stimulation (light grey area), others did not (dark grey area).

### Subjective evaluation of speech and voice (VAS pronunciation, SHI, VAS voice, VHI)

#### VAS pronunciation quality

**Fig. 5:**
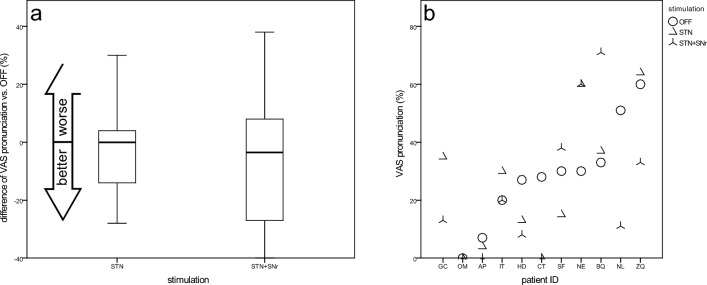
Visual analog scale for pronunciation—**a** boxplots of changes from STIM-OFF for stimulation groups and **b** scatterplot for individual patient outcomes (sorted by values for STIM-OFF)

The patients' subjective ratings of the quality of pronunciation were very heterogeneous. Six patients reported better values under at least one stimulation condition compared to DBS OFF, two rated the quality worse under both stimulations (Fig. 5b). Compared to OFF (28.6 ± 17.9), both, STN -DBS (25.8 ± 23.4) and STN + SNr-DBS (23.1 ± 24.6) revealed slightly, but not significantly improved results compared to DBS OFF (Fig. 5a).

#### Speech handicap index (SHI)

**Fig. 6:**
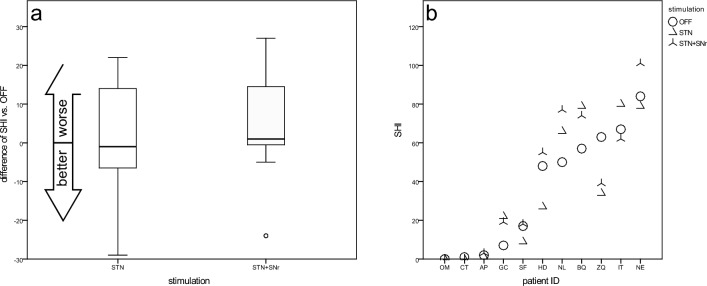
Speech Handicap Index (SHI)—**a** boxplots of changes from STIM-OFF for stimulation groups and **b** scatterplot for individual patient outcomes (sorted by values for STIM-OFF)

SHI results of PD patients in DBS OFF were scattered over the whole range. Related to the group average, both stimulation conditions had no influence on the SHI results. At the individual level, the SHI score improved in seven patients under at least one stimulation condition. In three patients they worsened under both stimulation conditions (Fig. 6b). Compared to DBS OFF (36.0 ± 31.1), STN –DBS (36.1 ± 33.7) revealed equal and STN + SNr-DBS (40.7 ± 35.3) worse SHI scores (Fig. 6a).

#### VAS voice quality

**Fig. 7:**
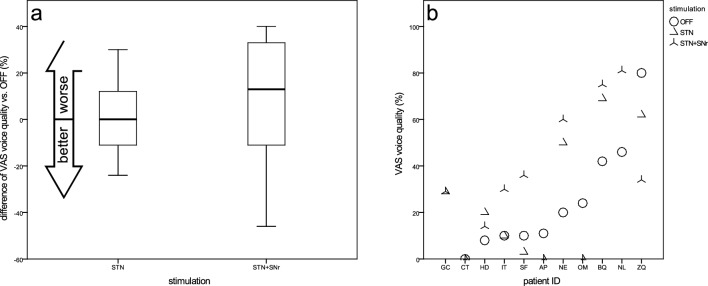
Visual analog scale for voice quality—**a** boxplots of changes from STIM-OFF for stimulation groups and **b** scatterplot for individual patient outcomes (sorted by values for STIM-OFF)

Most patients reported acceptable voice quality in STIM-OFF mode (25.1 ± 24.4), corresponding to a VAS score below 30%. No stimulation condition was found to be superior. In four patients, voice quality improved under at least one stimulation, and in three patients it worsened under both stimulations (Fig. 7b). On average, STN-DBS was nearly equivalent to DBS OFF and yielded better voice quality (24.3 ± 27.0) compared to STN + SNr-DBS (27.5 ± 26.3) (Fig. 7a).

#### Voice handicap index (VHI)

**Fig. 8:**
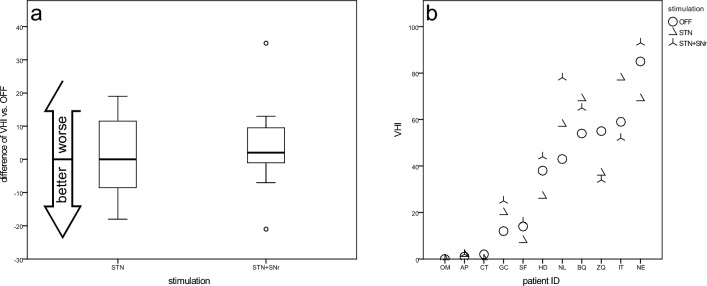
Voice Handicap Index (VHI)—**a** boxplots of changes from STIM-OFF for stimulation groups and **b** scatterplot for individual patient outcomes (sorted by values for STIM-OFF)

As shown in Fig. 8b, the VHI in the DBS OFF scattered over the entire range of the VHI. Overall, the two DBS conditions showed a heterogeneous effect on VHI scores. In six patients, the value improved under at least one stimulation, and in four patients, the results worsened under both stimulation conditions. Compared to DBS OFF (33.0 ± 28.8), STN-DBS (33.5 ± 30.4) produced similar results, while STN + SNr stimulation resulted in poorer outcomes (37.2 ± 32.3).

## Discussion

This study assessed the effects on speech and voice parameters of combined STN + SNr-DBS compared to conventional STN-DBS in PD patients. The strong correlation of stimulation condition and stimulation energy (TEED) might be a confounding factor in the interpretation of the observations. Results of this study which consider the stimulation condition have to be interpreted as results of the combined influence of stimulation condition and stimulation energy.

The objective speech parameters (mean F0, intonation, syllable rates, and reading time) were not significantly influenced by neither conventional nor combined stimulation. Interestingly, subjective self-perception often did not reflect objectively measured parameters of speech and voice function in this study. Both stimulation conditions did not influence objective voice quality significantly, but individual effects were observable. Subjective speech and voice quality parameters (VHI, SHI, and VAS pronunciation/voice) were not significantly changed by STN but—except for VAS pronunciation—deteriorated slightly, but not significantly under combined stimulation.

The values for intonation differ slightly compared to other studies in which the mean variation coefficient for STN stimulation in male patients was 2.3 semitones (equivalent to 13.7% in frequency) (Mate et al. 2012), or 2.6 semitones for healthy subjects and 2.0 semitones for PD patients (males and females) without DBS (Rusz et al. 2020). That means that the data of the present study show that the intonation values described by Rusz et al. as typical for Parkinson's disease (Rusz et al. 2020) are obtained only under STN-DBS. The values under STN + SNr mode as well as in STIM-OFF in our study are comparable to those of healthy controls in the study by Rusz et al.

To date, it is still unclear what specific effects DBS, and in particular the combined stimulation of STN + SNr, has on speech and voice production (Pinto et al. 2005). In contrast, the positive impact on motor symptoms in PD patients is proven and attention has been primarily focused on the effects of DBS on motor function. However, with 29% of PD patients reporting communication impairment as their most important limitation (Dashtipour et al. 2018), this should also be focused on. Proper speech and voice function are essential components of quality of life. Therefore, in contrast to other research areas, it is particularly important for the analysis of speech and voice quality to use not only objective parameters, but also the subjective perception.

Dysarthria is considered one of the most common side effects of STN-DBS and must be minimized by DBS programming. A meta-analysis reported stimulation-related dysarthria in nearly 10% of the patients (Kleiner-Fisman et al. 2006). However, in the literature, there is a lack of methodologically adequate studies with larger numbers of cases that have evaluated speech-specific (like AVQI) or voice-specific (like intonation) parameters.

Different troubleshooting options of DBS can be considered to improve side-effects like dysphagia or dysarthria. Previously, the use of low frequency stimulation with 60 Hz STN-DBS has been proven to be beneficial to ameliorate dysphagia, but with subsiding long-term effects (Xie et al. 2018). Other therapeutic approaches could be the application of bipolar stimulation or possibly short-pulse stimulation (Reich et al. 2015), when dysarthria is supposed to be a stimulation-induced side effect of unintended co-stimulation of the capsula interna.

The well-established and commonly used VHI shows that STN -DBS did not deteriorate subjective voice handicap. This is in line with Onder et al. (2023), but not with Tanaka et al. who found VHI worsening (Tanaka et al. 2015). This study is the first to compare conventional STN-DBS with combined STN + SNr-DBS and revealed that the VHI worsened slightly, but not significantly under combined stimulation.

To the best of our knowledge, there are no available data using the AVQI in DBS studies. The AVQI is a robust measure of dysphonia (hoarseness) that has been widely used in clinical practice and research. It correlates well with auditory-perceptual assessments of voice quality and has high test–retest reliability and sensitivity. Because of the combined use of continuous speech and sustained vocal phonation in this analysis, it is reasonable to use it in this context. Interestingly, all patients studied are outside the normophonic range in AVQI, regardless of DBS mode. This can most likely be interpreted as PD-associated hoarseness. Ramig et al. found jitter and shimmer values more than doubled in PD patients compared to a healthy control group (Ramig et al. 1988).

Overall, the male patients examined in the present study showed a higher speaking voice fundamental frequency (mean F0) both in STIM-OFF and under both DBS modes. Possible causes for this high fundamental frequency may include the PD itself or a side effect of DBS implantation (Tanaka et al. 2015). Thus, F0 variability reduced by hypokinesia is considered a major aspect of hypokinetic dysarthria (Skodda et al. 2014; Sapir 2014).

One important factor for the comparability with other studies is that there is no standardized or validated protocol for speech and voice diagnostics in PD patients (Defazio et al. 2016; Brabenec et al. 2017; Galaz et al. 2018). Thus, valid acoustic measurement procedures are often not specified and sometimes only subjective outcome parameters are used (Rusz et al. 2020). In particular, there is high variability in speech and voice parameters studied (Tsuboi et al. 2015). In addition, numerous other factors may influence measurements: disease duration, side effects of DBS surgery, patient motivation and agitation during voice recordings, training effects, medication side effects, time-of-day effects, small patient cohort, and other factors.

Although small, individual effects of both DBS conditions on speech and voice parameters were observed, they were non-significant and not relevant for daily life. In summary, no preference for one stimulation condition can be derived from the results in terms of patient speech and voice performance. Therefore, from a voice specialist's point of view, the DBS modalities investigated here are both safe for PD patients.

## Conclusion

The stimulation induced effects on speech and voice production are minor relative to the benefits of motor limitations improved by DBS for most patients. Our results imply that combined STN + SNr stimulation is safe in terms of patient’s speech and voice. However, there is no clear superior effect of STN + SNr-DBS compared to STN-DBS for best communication results. The individualized optimization of stimulation is the only way to improve the voice-related quality of life of specific patients.

## Data availability statement

The data supporting the findings of this study are available on request from the corresponding author. The data are not publicly available due to privacy or ethical restrictions.

## Supplementary Information

Below is the link to the electronic supplementary material.

Supplementary file1 (DOCX 638 KB)

## Data Availability

The data are available on request.

## References

[CR1] Ackermann H (2008) Cerebellar contributions to speech production and speech perception: psycholinguistic and neurobiological perspectives. Trends Neurosci 31:265–272. 10.1016/j.tins.2008.02.011 18471906 10.1016/j.tins.2008.02.011

[CR2] Baken RJ, Orlikoff RF (2000) Clinical measurement of speech and voice. Singular Thomson Learning

[CR3] Barsties von Latoszek B, Lehnert B (2018) Interne Validität des acoustic voice quality index version 03.01 und des acoustic breathiness index. Laryngo-Rhino-Otol 97:630–635. 10.1055/a-0596-7819 10.1055/a-0596-7819 29635679

[CR4] Barsties von Latoszek B, Lehnert B, Janotte B (2020) Validation of the acoustic voice quality index version 03.01 and acoustic breathiness index in German. J Voice 34:157.e17-157.e25. 10.1016/j.jvoice.2018.07.026 10.1016/j.jvoice.2018.07.026 30217485

[CR5] Baudouin R, Lechien JR, Carpentier L et al (2023) Deep brain stimulation impact on voice and speech quality in Parkinson’s disease: a systematic review. Otolaryngol Head Neck Surg 168:307–318. 10.1177/01945998221120189 36040825 10.1177/01945998221120189

[CR6] Berg M, Fuchs M, Wirkner K et al (2017) The speaking voice in the general population: normative data and associations to sociodemographic and lifestyle factors. J Voice 31:257.e13-257.e24. 10.1016/j.jvoice.2016.06.001 27370073 10.1016/j.jvoice.2016.06.001

[CR7] Boersma P, Weenink D (2021) Praat: doing Phonetics by Computer

[CR8] Brabenec L, Mekyska J, Galaz Z, Rektorova I (2017) Speech disorders in Parkinson’s disease: early diagnostics and effects of medication and brain stimulation. J Neural Transm (Vienna) 124:303–334. 10.1007/s00702-017-1676-0 28101650 10.1007/s00702-017-1676-0

[CR9] Dashtipour K, Tafreshi A, Lee J, Crawley B (2018) Speech disorders in Parkinson’s disease: pathophysiology, medical management and surgical approaches. Neurodegener Dis Manag 8:337–348. 10.2217/nmt-2018-0021 30223711 10.2217/nmt-2018-0021

[CR10] Defazio G, Guerrieri M, Liuzzi D et al (2016) Assessment of voice and speech symptoms in early Parkinson’s disease by the Robertson dysarthria profile. Neurol Sci 37:443–449. 10.1007/s10072-015-2422-8 26615536 10.1007/s10072-015-2422-8

[CR11] Deliyski DD, Shaw HS, Evans MK (2005) Adverse effects of environmental noise on acoustic voice quality measurements. J Voice 19:15–28. 10.1016/j.jvoice.2004.07.003 15766847 10.1016/j.jvoice.2004.07.003

[CR12] Deuschl G, Schade-Brittinger C, Krack P et al (2006) A randomized trial of deep-brain stimulation for Parkinson’s disease. N Engl J Med 355:896–908. 10.1056/NEJMoa060281 16943402 10.1056/NEJMoa060281

[CR13] Deuschl G, Paschen S, Witt K (2013) Chapter 10 - Clinical outcome of deep brain stimulation for Parkinson’s disease. In: Lozano AM, Hallett M (eds) Handbook of clinical neurology. Elsevier, Amsterdam, pp 107–128 10.1016/B978-0-444-53497-2.00010-3 24112889

[CR14] Galaz Z, Mekyska J, Zvoncak V, et al (2018) Changes in phonation and their relations with progress of Parkinson’s disease

[CR15] Hidding U, Gulberti A, Horn A et al (2017) Impact of combined subthalamic nucleus and substantia Nigra stimulation on neuropsychiatric symptoms in Parkinson’s disease patients. Parkinsons Dis 2017:7306192. 10.1155/2017/7306192 28246572 10.1155/2017/7306192 PMC5299199

[CR16] Ho AK, Iansek R, Marigliani C et al (1999) Speech impairment in a large sample of patients with Parkinson’s disease. Behav Neurol 11:131–137 22387592

[CR17] Jürgens U (2002) Neural pathways underlying vocal control. Neurosci Biobehav Rev 26:235–258. 10.1016/s0149-7634(01)00068-9 11856561 10.1016/s0149-7634(01)00068-9

[CR18] Kleiner-Fisman G, Herzog J, Fisman DN et al (2006) Subthalamic nucleus deep brain stimulation: summary and meta-analysis of outcomes. Mov Disord 21(Suppl 14):S290-304. 10.1002/mds.20962 16892449 10.1002/mds.20962

[CR19] Maryn Y, Corthals P, Van Cauwenberge P et al (2010) Toward improved ecological validity in the acoustic measurement of overall voice quality: combining continuous speech and sustained vowels. J Voice 24:540–555. 10.1016/j.jvoice.2008.12.014 19883993 10.1016/j.jvoice.2008.12.014

[CR20] Mate MA, Cobeta I, Jiménez-Jiménez FJ, Figueiras R (2012) Digital voice analysis in patients with advanced Parkinson’s disease undergoing deep brain stimulation therapy. J Voice 26:496–501. 10.1016/j.jvoice.2011.03.006 21704492 10.1016/j.jvoice.2011.03.006

[CR21] Mayer J (2021) Heuristic algorithm to detect syllable boundaries. In: praatpfanne.lingphon.net. https://praatpfanne.lingphon.net/downloads/syllable_candidates.txt. Accessed 28 Oct 2021

[CR22] Miller N, Noble E, Jones D, Burn D (2006) Life with communication changes in Parkinson’s disease. Age Ageing 35:235–239. 10.1093/ageing/afj053 16540492 10.1093/ageing/afj053

[CR23] Moreau C, Defebvre L, Destée A et al (2008) STN-DBS frequency effects on freezing of gait in advanced Parkinson disease. Neurology 71:80–84. 10.1212/01.wnl.0000303972.16279.46 18420482 10.1212/01.wnl.0000303972.16279.46

[CR24] Onder H, Bahtiyarca ZT, Comoglu S (2023) Subjective assessments of voice in Parkinson’s disease subjects with and without STN-DBS therapy. Ann Indian Acad Neurol 26:491–495. 10.4103/aian.aian_1_23 37970309 10.4103/aian.aian_1_23 PMC10645232

[CR25] Pflug C, Nienstedt JC, Gulberti A et al (2020) Impact of simultaneous subthalamic and nigral stimulation on dysphagia in Parkinson’s disease. Ann Clin Transl Neurol 7:628–638. 10.1002/acn3.51027 32267102 10.1002/acn3.51027 PMC7261764

[CR26] Pinto S, Ozsancak C, Tripoliti E et al (2004) Treatments for dysarthria in Parkinson’s disease. Lancet Neurol 3:547–556. 10.1016/S1474-4422(04)00854-3 15324723 10.1016/S1474-4422(04)00854-3

[CR27] Pinto S, Gentil M, Krack P et al (2005) Changes induced by levodopa and subthalamic nucleus stimulation on parkinsonian speech. Mov Disord 20:1507–1515. 10.1002/mds.20601 16037917 10.1002/mds.20601

[CR28] Ramig LA, Scherer RC, Titze IR, Ringel SP (1988) Acoustic analysis of voices of patients with neurologic disease: rationale and preliminary data. Ann Otol Rhinol Laryngol 97:164–172. 10.1177/000348948809700214 2965542 10.1177/000348948809700214

[CR29] Reich MM, Steigerwald F, Sawalhe AD et al (2015) Short pulse width widens the therapeutic window of subthalamic neurostimulation. Ann Clin Transl Neurol 2:427–432. 10.1002/acn3.168 25909087 10.1002/acn3.168 PMC4402087

[CR30] Rinkel RN, Verdonck-de Leeuw IM, van Reij EJ et al (2008) Speech Handicap Index in patients with oral and pharyngeal cancer: better understanding of patients’ complaints. Head Neck 30:868–874. 10.1002/hed.20795 18302270 10.1002/hed.20795

[CR31] Rosen CA, Lee AS, Osborne J et al (2004) Development and validation of the voice handicap index-10. Laryngoscope 114:1549–1556. 10.1097/00005537-200409000-00009 15475780 10.1097/00005537-200409000-00009

[CR32] Rusz J, Tykalova T, Ramig LO, Tripoliti E (2020) Guidelines for speech recording and acoustic analyses in dysarthrias of movement disorders. Mov Disord. 10.1002/mds.28465 33373483 10.1002/mds.28465

[CR33] Sapir S (2014) Multiple factors are involved in the dysarthria associated with Parkinson’s disease: a review with implications for clinical practice and research. J Speech Lang Hear Res 57:1330–1343. 10.1044/2014_JSLHR-S-13-0039 24686571 10.1044/2014_JSLHR-S-13-0039

[CR34] Schuepbach WMM, Rau J, Knudsen K et al (2013) Neurostimulation for Parkinson’s disease with early motor complications. N Engl J Med 368:610–622. 10.1056/NEJMoa1205158 23406026 10.1056/NEJMoa1205158

[CR35] Schulz GM, Grant MK (2000) Effects of speech therapy and pharmacologic and surgical treatments on voice and speech in Parkinson’s disease: a review of the literature. J Commun Disord 33:59–88. 10.1016/s0021-9924(99)00025-8 10665513 10.1016/s0021-9924(99)00025-8

[CR36] Skodda S, Grönheit W, Schlegel U et al (2014) Effect of subthalamic stimulation on voice and speech in Parkinson’s disease: for the better or worse? Front Neurol 4:218. 10.3389/fneur.2013.00218 24454305 10.3389/fneur.2013.00218 PMC3888994

[CR37] Steigerwald F, Pötter M, Herzog J et al (2008) Neuronal activity of the human subthalamic nucleus in the parkinsonian and nonparkinsonian state. J Neurophysiol 100:2515–2524. 10.1152/jn.90574.2008 18701754 10.1152/jn.90574.2008

[CR38] Tanaka Y, Tsuboi T, Watanabe H et al (2015) Voice features of Parkinson’s disease patients with subthalamic nucleus deep brain stimulation. J Neurol 262:1173–1181. 10.1007/s00415-015-7681-z 25712544 10.1007/s00415-015-7681-z

[CR39] Tsuboi T, Watanabe H, Tanaka Y et al (2015) Distinct phenotypes of speech and voice disorders in Parkinson’s disease after subthalamic nucleus deep brain stimulation. J Neurol Neurosurg Psychiatry 86:856–864. 10.1136/jnnp-2014-308043 25280914 10.1136/jnnp-2014-308043

[CR40] Weiss D, Walach M, Meisner C et al (2013) Nigral stimulation for resistant axial motor impairment in Parkinson’s disease? A randomized controlled trial. Brain 136:2098–2108. 10.1093/brain/awt122 23757762 10.1093/brain/awt122 PMC3692032

[CR41] Xie T, Bloom L, Padmanaban M et al (2018) Long-term effect of low frequency stimulation of STN on dysphagia, freezing of gait and other motor symptoms in PD. J Neurol Neurosurg Psychiatry 89:989–994. 10.1136/jnnp-2018-318060 29654112 10.1136/jnnp-2018-318060

