# Supplementary material for: Effect of subthalamic and nigral deep brain stimulation on speech and voice in Parkinson’s patients
Source: J Neural Transm (Vienna). 2024 Nov 28;132(3):419–29. doi: 10.1007/s00702-024-02860-5 (PMC11870922; doi:10.1007/s00702-024-02860-5)
Supplement: Supplementary file 1 — Supplementary file1 (DOCX 638 KB) [file 702_2024_2860_MOESM1_ESM.docx]

## Supplement

Patient characteristics

|  |  |  |  |  |  | STN-DBS parameters | Combined STN+SNr-DBS parameters | X, Y, Z coordinates |
| --- | --- | --- | --- | --- | --- | --- | --- | --- |
| Case  Gender  Age | Age at onset | Disease duration [years] | Time with DBS [months] | LEDD [mg] | DBS  System | Left electrode (1. row)  Right electrode (2. row) | Left electrode (1. row)  Right electrode (2. row) | Left electrode  (1. row)  Right electrode (2. row) |
| 1 M 61 | 38 | 23 | 54 | 1150 | MD | 1- 2- G+, 3.5V, 60 µs, 125 Hz  9- 10- G+, 2.7 V, 60 µs, 125 Hz | 1- 2- G+, 3.5V, 60 µs, 125 Hz; 0- G+, 2.0 V, 60 µs, 125 Hz  9- 10- G+, 2.7 V, 60 µs, 125 Hz, 8- G+, 2.0 V, 60 µs, 125 Hz | 10.9, 2.2, 4.7  10.5, 3.8, 4.7 |
| 2 M 63 | 40 | 23 | 105 | 860 | MD | 1- G+, 1.9 V, 60 µs, 125 Hz; 2- G+, 2.9 V, 60 µs, 125 Hz  9- G+, 1.9V, 60 µs, 125 Hz; 10- G+, 3.3 V, 60 µs, 125 Hz | 2- G+, 2.9 V, 60 µs, 125 Hz; 1- 0- G+, 1.9 V (1.5 V), 60 µs, 125 Hz  10- G+, 3.3 V, 60 µs, 125 Hz; 8- 9- G+, 1.9 V (1.5 V) 60 µs, 125 Hz | 11.2 1.9, 5.6  8.3, 5.5, 4 |
| 3 M 56 | 47 | 9 | 36 | 880 | MD | 1+ 2- G+ 2.2 V, 60 µs, 125 Hz  10- G+, 4.3 V, 60 µs, 125 Hz | 2-G+, 2.2V, 60 µs, 125 Hz; 0- G+, 1.0 V, 60 µs, 125 Hz  10- G+, 4.3V, 60 µs, 125 Hz, 8- G+, 1.0 V, 60 µs, 125 Hz | 9.5, 2.8, 6.4  11.2, 1.4, 7.2 |
| 4 M 67 | 51 | 16 | 60 | 600 | MD | 1- G+, 1.5 V, 60 µ, 125 Hz  9- 10- G+, 3.9 V, 60 µs, 125 Hz | 1- G+, 1.5 V, 60 µ, 125 Hz; 0- G+, 2.0 V, 60 µs, 125 Hz  9-10- G+, 3.9 V, 60 µs, 125 Hz; 8- G+, 2.0 V, 60 µs, 125 Hz | 9.6, 4.7, 6.6  11.7, 3.1, 3.2 |
| 5 M 65 | 56 | 9 | 9 | 300 | MD | 1- G+, 2.8 V, 60 µs, 125 Hz  9- G+, 3.0 V, 60 µs, 125 Hz | 1-G+, 2.8 V, 60 µs, 125 Hu; 0- G+, 1.5 V, 60 µs, 125 Hz  9-G+, 3.0 V, 60 µs, 125 Hz; 8- G+, 1.5 V, 60 µs | 10.9, 1.4, 7.7  11.1, 2.7, 6.7 |
| 6 M 74 | 65 | 9 | 9 | 360 | MD | 1- G+, 2.7 V, 130 Hz  9- G+, 2.6 V, 60 µs, 130 Hz | 1- G+, 2.7 V, 60 µs, 125 Hz; 0- G+, 1.5 V, 60 µs, 125 Hz  9- G+, 2.9 V, 60 µs, 125 Hz; 8- G+, 1.5 V, 60 µs, 125 Hz | 10.7, 2.6, 4.9  10.2, 2.5, 4.5 |
| 7 M 51 | 42 | 9 | 15 | 900 | BS | 2- 30%, 3- 70%, 3,4 mA, 60 µs, 125 Hz  10- 20%, 11- 80%, 4,0 mA, 60 µs, 125 Hz | 1- 23%, 2- 23%, 3- 54%, 4.4 mA, 60 µs, 125 Hz  9- 20%, 10- 16%, 11- 64%, 5.0 mA, 60 µs, 125 Hz | 8.81, 3.38, 7.37  7.04, 4.28, 6.41 |
| 8 M 57 | 50 | 7 | 18 | 580 | BS | 3- 70%, 4- 30%, 4,5 mA, 60 µsec, 130 Hz  12- 100% 3,8 mA, 60 µsec, 130 Hz | 3- 61%, 4- 26%, 1- 13%, 5,2 mA, 60 µsec, 130 Hz  12- 85%, 9- 15%, 4,5 mA, 60 µsec, 130 Hz | 11.85, 3.37, 6.09  11.63, 2.69, 5.9 |
| 9 M 71 | 60 | 11 | 13 | 950 | MD | 1- G+, 3,5 V, 60 µsec, 125 Hz  9- G+, 2,7 V, 60 µsec, 125 Hz | 1- G+, 3,5 V, 60 µsec, 125 Hz, 0- G+, 1,0 V, 60 µsec, 125 Hz  9- G+, 2,7 V, 60 µsec, 125 Hz; 8- G+, 1,0 V, 60 µsec, 125 Hz | 11.34, 2.18, 6.24  12.23, 0.2, 5.22 |
| 10 M 66 | 54 | 13 | 6 | 1000 | MD | 1- G+, 3.8 V, 60 µsec, 130 Hz  9- G+, 3.6 V, 60 µz 130 Hz | 1- 3.8 V 60 µsec 125 Hz 0- 1.0 V 60 µsec, 125 Hz  9- G+ 3.6 V, 60 µsec, 125 Hz 8- G+ 1,0 V 60 µsec, 125 Hz | 11.24, 6.46, 6.6  10.53, 4.23, 5.09 |
| 11 W 66 | 56 | 10 | 5 | 1245 | BS | 5-6-7- (Ring)G+ 2.2 mA, 60 µsec, 130 Hz  13-14-15- (Ring) G+, 2.4 mA 60 µsec, 130 Hz | 5- (23%) 6- (23%) 7- (23%) 1- (31%) G+ 2.9 mA, 60 µsec, 130 Hz  13-(24%) 14- (23%) 15-(23%) 9-(30%) G+, 3.1mA, 60 µsec, 130 Hz | 10.86, 2.51, 5.69  10.42, 0.44, 5.23 |
| Mn 63.4 | 50.8 | 12.6 | 30.0 | 802.3 |  |  |  |  |
| SD 6.7 | 8.5 | 5.7 | 31.4 | 305.8 |  |  |  |  |

**Tab. S 1** Disease duration [years]" is calculated from the date of the first diagnosis to the date of baseline measurement of the experiment. “DBS parameters” include: Active contacts, amplitude (volts or mA), pulse width (microseconds) and stimulation frequency (Hz), for the left and right electrode, respectively. Electrode coordinates are given as mm lateral to the midline (X), posterior to the mid-commissural point (Y) and inferior to the intercommissural plane (Z). Note that the deepest contacts were contact 0 and 8 (Medtronic) or contact 1 and 9 (Boston Scientific). LEDD, levodopa equivalent daily dose; ME, Medtronic. BS, Boston Scientific.

Location of DBS electrode contacts


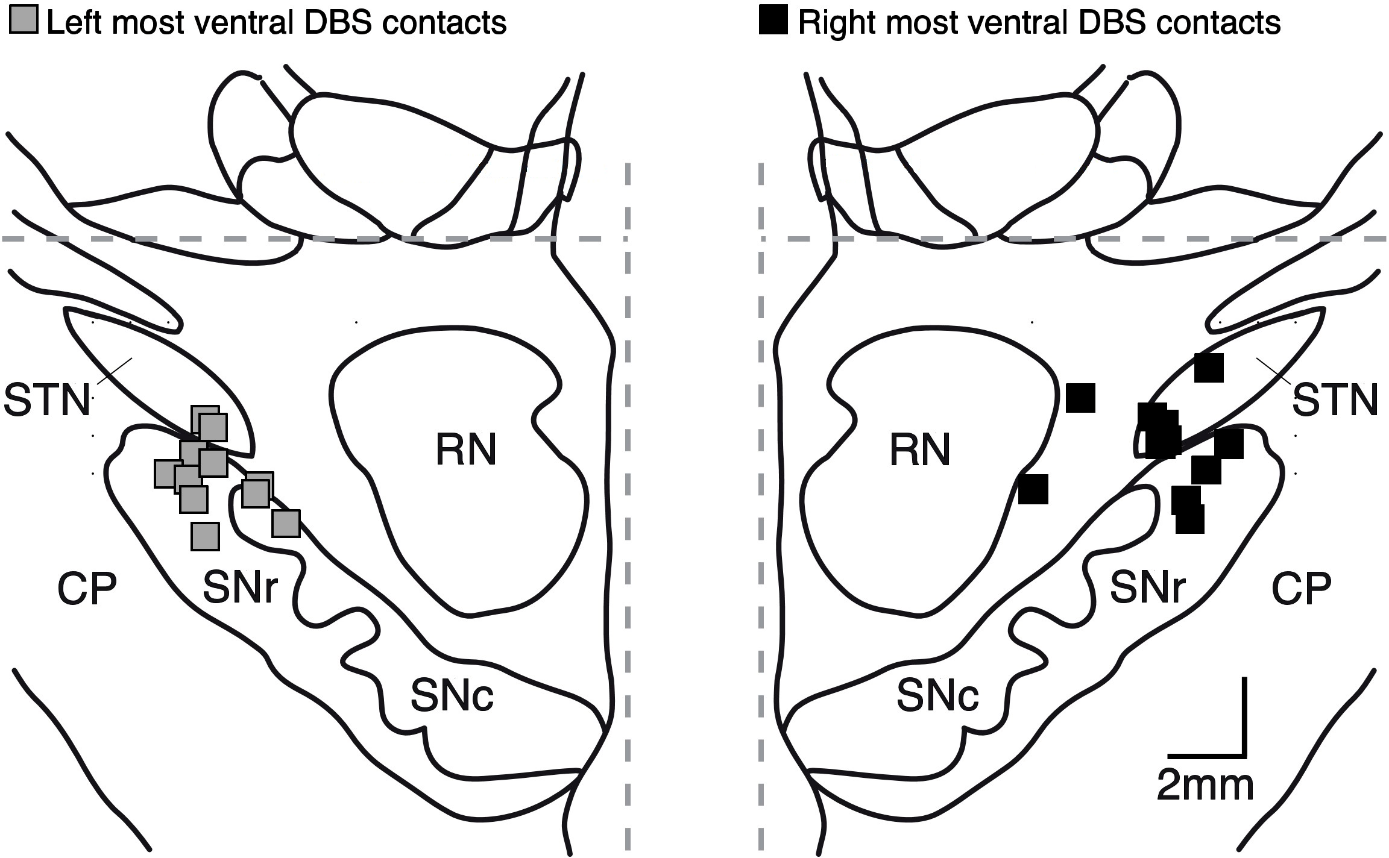


**Fig. S 1** Location of DBS electrode contacts. The dashed gray lines denote midline and AC-PC level, respectively. Abbreviations: STN, subthalamic nucleus; SNr, substantia nigra, pars reticulata; SNc, substantia nigra, pars compacta; CP, cerebral peduncle; RN, red nucleus.

Results – additional material

**Fig. S 2** Correlation of intonation (variation coefficient of F0) with TEED.

#### Syllable rate [pataka]

**Fig. S 3** Syllable rate for [pataka] – (a) boxplots of changes from STIM-OFF for stimulation groups and (b) scatterplot for individual patient outcomes (sorted by values for STIM-OFF).

Compared to OFF mode (5.8 ± 1.5), the mean syllable rate for [pataka] decreases under STN stimulation (5.7 ± 1.3) and improves under STN+SNr (6.1 ± 0.9) stimulation (Fig. S 3a), but the means of both stimulations are not significantly different (CI: -0.6, 0.4, t-test for connected samples). When analyzed intra-individually, six patients showed improvement under at least one stimulation and three patients showed deterioration under both stimulations (Fig. S 3b).

#### Syllable rate [pa]

**Fig. S 4** Syllable rate for [pa] – (a) boxplots of changes from STIM-OFF for stimulation groups and (b) scatterplot for individual patient outcomes (sorted by values for STIM-OFF).

Compared to OFF mode (6.1 ± 1.0), the mean syllable rate for [pa] decreases under STN stimulation (5.7 ± 1.2) and improves under STN+SNr stimulation (6.2 ± 1.2) (Fig. S 4a). There are three patients with an improvement with at least one stimulation and two patients with a worse rate with both stimulations (Fig. S 4b).

#### Reading time (RT)

**Fig. S 5** Reading time – (a) boxplots of changes from STIM-OFF for stimulation groups and (b) scatterplot for individual patient outcomes (sorted by values for STIM-OFF).

The duration of reading the passage was in STIM-OFF was 49.5 s (± 15.2). The reading was slightly quicker for STN stimulation (46.1 ± 10.4) and equal for STN+SNr stimulation (49.6 ± 15.8) (Fig. S 5a). In three patients, RT prolonged slightly under both types of stimulation. In six patients, the RT improved at least under one type of stimulation (Fig. S 5b).
